# Changes in health worker knowledge and motivation in the context of a quality improvement programme in Ethiopia

**DOI:** 10.1093/heapol/czab094

**Published:** 2021-08-10

**Authors:** Matthew Quaife, Abiy Seifu Estafinos, Dorka Woldesenbet Keraga, Julia Lohmann, Zelee Hill, Abiyou Kiflie, Tanya Marchant, Josephine Borghi, Joanna Schellenberg

**Affiliations:** Faculty of Public Health and Policy, London School of Hygiene and Tropical Medicine, 15-17 Tavistock Place, London WC1H 9SH, UK; School of Public Health, Addis Ababa University, Addis Ababa, Ethiopia; School of Public Health, Addis Ababa University, Addis Ababa, Ethiopia; Faculty of Public Health and Policy, London School of Hygiene and Tropical Medicine, 15-17 Tavistock Place, London WC1H 9SH, UK; Institute for Global Health, University College London, Gower Street, London WC1E 6BT, UK; Institute for Healthcare Improvement, Addis Ababa, Ethiopia; Faculty of Infectious and Tropical Diseases, London School of Hygiene and Tropical Medicine, Keppel Street, London WC1E 7HT, UK; Faculty of Public Health and Policy, London School of Hygiene and Tropical Medicine, 15-17 Tavistock Place, London WC1H 9SH, UK; Faculty of Infectious and Tropical Diseases, London School of Hygiene and Tropical Medicine, Keppel Street, London WC1E 7HT, UK

**Keywords:** Quality improvement, health worker motivation, health worker knowledge, mixed methods, Ethiopia

## Abstract

A knowledgeable and motivated workforce is critical for health systems to provide high-quality services. Many low- and middle-income countries face shortages in human resources and low health worker motivation but are also home to a burgeoning number of quality improvement (QI) programmes. This study evaluates whether and how motivation and clinical knowledge in three cadres of health workers changed in the context of a QI programme for maternal and newborn health in Ethiopia. This mixed-methods study used a pre–post comparison group design with matched comparison areas. We interviewed 395 health workers at baseline in April 2018 and 404 at endline in June 2019 from seven districts (woredas) with QI and seven comparison woredas. Three cadres were interviewed: health extension workers, facility-based skilled midlevel maternal and newborn care providers, and non-patient-facing staff. A qualitative component sought to triangulate and further elucidate quantitative findings using in-depth interviews with 22 health workers. Motivation was assessed quantitatively, exploratory factor analysis was used to categorize motivation dimensions, and regression-based difference-in-difference analyses were conducted. Knowledge was assessed through a clinical vignette. Qualitative data were analysed in a deductive process based on a framework derived from quantitative results. Although knowledge of the QI programme was high (79%) among participants from QI woreda at endline, participation in QI teams was lower (56%). There was strong evidence that health worker knowledge increased more in areas with QI than comparison areas. Three motivation dimensions emerged from the data: (1) ‘helping others’, (2) ‘pride and satisfaction’ and (3) ‘external recognition and support’. We found strong evidence that motivation across these factors improved in both QI and comparison areas, with weak evidence of greater increases in comparison areas. Qualitative data suggested the QI programme may have improved motivation by allowing staff to provide better care. This study suggests that although QI programmes can increase health worker knowledge, there may be little effect on motivation. Programme evaluations should measure a wide range of outcomes to fully understand their impact.

**Key messages**

- A knowledgeable and motivated health workforce is important for health systems to provide high-quality care. QI programmes seek to improve healthcare quality through a variety of mechanisms.
- We find that a QI programme was associated with an increase in health worker knowledge, but not associated with an increase in health worker motivation. Qualitative data suggest that motivation can be improved through increasing health worker skills.
- It is critical that programme evaluations measure a range of outcomes to understand their impact on patients and health workers.

## Introduction

Present, productive, and skilled health workers are key to a well-functioning health system (World Health Organization, 2010), and equitable access to an effective health system is needed to meet the Sustainable Development Goals (Lassi *et al.*, 2016). Many low- and middle-income countries do not meet international targets on health worker recruitment and retention (World Health Organization, 2016). Yet, even when healthcare workers are present, health worker knowledge and motivation play a critical role in performance and quality of care (Franco *et al.*, 2002; Mosadeghrad, 2014). Both are key focus areas of policies around human resources for health (World Health Organization, 2016), an important pillar of the health system.

In the Millennium Development Goals era between 1990 and 2015, Ethiopia reduced maternal and under-5 mortality by 70% (Countdown to 2015, 2015, Central Statistical Agency (CSA) [Ethiopia] and ICF, 2016), yet maternal and neonatal mortality remained high at 401 deaths per 100 000 live births and 55 deaths per 1 000 live births in 2019, respectively (World Health Organization, 2019). Supply- and demand-side factors contributed to this. For example, just 68% of pregnant women complete four antenatal care visits, and fewer than half of mothers receive a clinical check-up after delivery (Central Statistical Agency (CSA) [Ethiopia] and ICF, 2016; Kruk *et al.*, 2018). The supply side faces considerable constraints, which are important for this study. Ethiopia experiences significant issues in the stock, retention, distribution and performance of health workers with performance inhibited by low salaries, a lack of access to training and poor facility infrastructure (Dagne *et al.*, 2015). Ethiopia’s maternal and child health workforce is composed of different cadres, which we broadly define here as (1) community health workers, known as health extension workers (HEWs); (2) facility-based, skilled midlevel maternal and newborn care providers, hereafter referred to as midlevel care providers, such as midwives or anaesthetic practitioners; (3) medical doctors and surgeons; and (4) non-patient-facing staff (Wang *et al.*, 2016; Arora *et al.*, 2020)—due to the small number of medical doctors and surgeons, we pool (2) and (3) together in this study.

The focus of this study is a quality improvement (QI) programme implemented in Ethiopia. The ‘Ethiopia Health Care Quality Initiative’ was implemented by the Ethiopian Federal Ministry of Health with support from the Institute for Healthcare Improvement between 2017 and 2020. Although there is no unambiguous definition of what constitutes a QI programme (Jones *et al.*, 2019), common features include encouraging leadership by frontline health service providers who identify problems and propose pragmatic solutions, followed by reflection and evaluation to assess if changes affect practice (Margolis and Boffito, 2015; Jones *et al.*, 2019). Over the last decade, the number of QI programmes implemented in country health systems across the income spectrum has increased markedly (Powell *et al.*, 2009; Dixon-Woods and Martin, 2016; Wagenaar *et al.*, 2017). Evaluations of QI programmes to-date have assessed their impact on healthcare quality or patient outcomes, with less focus on their impact on health worker motivation and knowledge (Knudsen *et al.*, 2019). The evaluations of some pay-for-performance programmes have sought to measure their impact on motivation; however, in seeking to improve quality through financial incentives these have a much narrower focus than many QI programmes (Lohmann *et al.*, 2016). Such programmes explicitly identify health worker motivation as a key mechanism to achieve impact by encouraging providers to exert more effort in return for financial incentives (Borghi *et al.*, 2018) and have been found to have little or no impact on health worker motivation (Ogundeji *et al.*, 2016; Shen *et al.*, 2017). More broadly, a recent systematic review found that strategies to improve health worker performance had varying effectiveness in different cadres (Rowe *et al.*, 2018), suggesting that it is important to understand heterogeneity in programme impact by cadre.

This study fills two key gaps in the literature on health worker knowledge and motivation. First, to our knowledge no study to-date has assessed how QI programmes affect health worker motivation and knowledge. Second, previous studies measuring health worker motivation in Ethiopia have been limited by focusing on midlevel hospital-based health workers in small geographical areas (Dagne *et al.*, 2015; Hotchkiss *et al.*, 2015; Weldegebriel *et al.*, 2016; Selamu *et al.*, 2017). In particular, this work has largely omitted the cadre of HEWs who provide the majority of maternal and child care and are a large part of the health workforce—21% of recurrent health expenditure in 2010/11 was spent on HEW salaries (Wang *et al.*, 2016).

**Figure 1.**
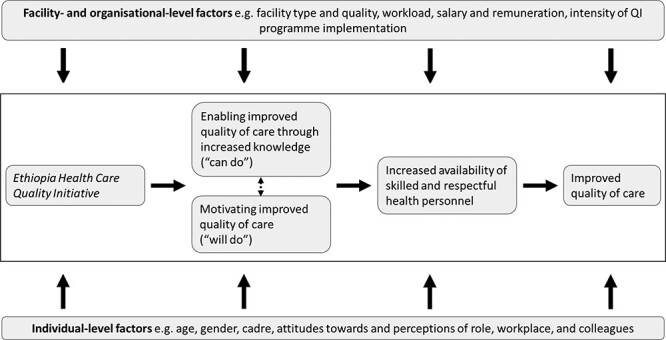
Theory of change.

This study used quantitative and qualitative data to evaluate whether and how the Ethiopia Health Care Quality Initiative affected health worker knowledge and motivation, and if effects differed by cadre. Our hypothesized mechanisms of actions are summarized in Figure 1 demonstrating our *a priori* conceptualization of how increased knowledge and motivation act in concert to improve quality of care. We assume that the extent to which the QI programme engenders improved quality of care is dependent on health workers being motivated by the QI programme and that motivation and quality of care are influenced by knowledge. We also assume that knowledge and motivation are influenced by a range of other factors at the individual and organizational levels. *A priori*, the relationship between knowledge and motivation was not clear—represented by a two-way arrow here. Specific hypotheses of how programme activities of training, mentorship, feedback and efficiency targets may affect motivation and knowledge are detailed in Supplementary file 1.

## Methods

### The QI programme

The QI programme was co-designed by Institute for Healthcare Improvement (IHI) and the Ethiopian Federal Ministry of Health, and implementation was accompanied by an impact and process evaluation, of which this study is one element (Hagaman *et al.*, 2020). In total, the programme was implemented in 19 woredas (districts) across four regions: seven in Oromia; five in Amhara; five in Southern Nations, Nationalities, and Peoples’ Region (SNNPR) and two in Tigray. The programme was later implemented in pastoralist Afar; however, this different context is outside the scope of this study. It sought to develop a national healthcare quality improvement strategy, build QI capacity at all health system levels and facilitate the introduction of district QI teams (‘collaboratives’) involving healthcare providers and managers. The programme formed collaboratives of multidisciplinary teams from all facilities in each woreda, and each facility team had between four and seven members comprising facility managers, physicians, nurses and midwives from the health centre and its associated health posts, health data officers and HEWs. Collaboratives lasted for around 15 months, with four half-week learning sessions separated by three action periods, and teams supported by external mentors from IHI and the Federal Ministry of Health. Further information on the programme and collaboratives is provided in Supplementary file 1. This study analyses data from the ‘test of scale’ phase of the QI programme, which expanded QI activities to a greater number of woredas, building on an earlier intensive ‘prototype’ phase in four woredas.

### Study design

We used a pre–post comparison group design, matching non-QI woredas to QI programme woreda using maternal and child health utilisation data from Demographic and Household Surveys (DHS). We used mixed methods, combining a repeated quantitative survey with supporting in-depth qualitative interviews—the purpose of the quantitative work was to measure motivation over time in QI and non-QI areas, whilst the purpose of the qualitative work was to validate quantitative measures and help understand the mechanisms of any impact observed in quantitative work. The quantitative component comprised a survey conducted with a cadre-stratified sample of randomly chosen HEWs, midlevel care providers and non-patient-facing staff. The survey sought to quantify motivation through a range of indicators and motivation domains, and the same tool was used at baseline and endline. We attempted to contact all baseline respondents to participate at endline. Because the QI programme sought to foster a culture of quality improvement in programme areas, we analysed data from all participants in QI programme and comparison areas, regardless of their participation in QI collaboratives or other QI activities. The qualitative component was conducted in between baseline and endline quantitative data collection, after a descriptive analysis of baseline data. We conducted semi-structured interviews with healthcare workers who were interviewed at baseline to triangulate and further expand quantitative findings and to capture other dynamics or factors which were not included in quantitative tools.

In seeking to measure the overall level of motivation that drives health worker behaviour, we take a ‘motivation intensity’ approach. Pinder defines motivation as a ‘set of energetic forces that originate both within as well as beyond an individual’s being, to initiate work-related behaviour and to determine its form, direction, intensity, and duration’ p. 11 (Pinder, 2014). The ‘motivation intensity’ approach assumes that health workers trade off time and effort with needs for rest, leisure and family time and therefore do not perform as well as possible, given their skill level and working conditions (Leonard *et al.*, 2013; Eichler and Levine, 2009). We hypothesize that the QI programme in this study could redirect health worker effort towards gaining knowledge, align health worker preferences with improved quality of care, and therefore improve knowledge and motivation (Lohmann *et al.*, 2016).

### Tool development

To measure health worker knowledge we used a clinical vignette. The vignette presented patient-facing respondents with a hypothetical scenario of a pregnant woman seeking antenatal care for the first time, and asked (1) what history questions they would ask, (2) what examinations and investigations they would perform, and (3) what drugs or supplies they would provide. The tool was taken from the World Bank Impact Evaluation Toolkit (World Bank, 2012) reviewed by one doctor and one midwife based in the UK, and one care provider in Ethiopia. The tool and possible responses are shown in Supplementary file 2.

We adapted a quantitative tool to measure motivation which was developed and validated among community health workers in Uganda (16), making minor changes to wording to suit the Ethiopian context. This initial tool consisted of 17 questions, and we added eight questions based on the World Bank Impact Evaluation Toolkit to explore extrinsic motivating factors in more depth. Finally, with input from senior staff implementing the QI programme we added five further questions around activities which were part of the programme, relating to training and recognition for doing a good job. In addition, we simply asked participants how they would rate their motivation at present. The motivation questions from the final tool are shown in Table 1. A target sample size of 50 respondents per region was chosen in line with rules of thumb from previous health worker motivation studies which indicated a minimum sample of 50 was sufficient for exploratory factor analysis (Lohmann *et al.*, 2016).

**Table 1.** Motivation questions included in survey

Variable wording
My work is important because I help people
As long as I can do what I enjoy, I’m not that concerned about exactly what income or awards I earn
I am respected in my community for the work I do
I am strongly motivated by the income I can earn at work
To be motivating, hard work must be rewarded with more status and money
My salary accurately reflects my skills and workload
I intend to stop working in this role in the next 12 months
I am proud of the work I do
In general, I am satisfied with my role
I gain knowledge from being in this role
Training sessions that I attend are worthwhile and add benefit to my career path
At the moment I don’t feel like working as hard as I can
I feel like performing the duties required of me
I am strongly motivated by the recognition I get from other people
It is important that I do a good job so that the health system works well
My job makes me feel good about myself
I feel it is not so important doing a good job if nobody else knows about it
I am willing to do more than is asked of me in my role
Sometimes I don’t understand why I am asked to do certain things, but I do them anyway
The system of choosing who attends training sessions is fair
I do not have enough opportunities to attend training sessions to develop my career
I am keenly aware of the career goals I have set for myself
If I do well at work, I will achieve my goals
I am proud to be working in my role
I feel committed to my role
The health system provides everything I need to do my job properly
I can solve most problems I have at work if I work hard
Suggestions made by people like me on how to improve their work are usually ignored by supervisors
My supervisors and managers are supportive of me
I can complete all of the work I am expected to do
‘Overall motivation intensity was measured through the following question, and not included in the factor analysis:’ How would you rate your overall motivation at your current work? ‘Possible responses were’ Excellent (=1), very good (=2), good (=3), fair (=4) and poor (=5)

Notes: All variables had Likert scale response options where 1 = strongly agree, 2 = agree, 3 = neutral, 4 = disagree and 5 = strongly disagree. Variables were chosen through examination of existing motivation measurement surveys and discussion among investigators.

Qualitative in-depth interview guides were initially developed using key themes reported by other health worker motivation studies in Ethiopia (Dagne *et al.*, 2015; Weldegebriel *et al.*, 2016; Selamu *et al.*, 2017). We used descriptive baseline quantitative data to refine guides to explore topics arising from quantitative work and to understand emergent themes more deeply. Guides were then finalized through discussion among researchers, with input from IHI staff and minor edits made during interviewer training.

### Sampling and data collection

#### Woreda selection and matched comparison

Using a random number generator, we randomly selected one QI programme woreda per region from the six QI woreda in Oromia, five in Amhara, four in SNNPR and two in Tigray. We added one additional randomly selected woreda in Amhara because the randomly chosen woreda would not have yielded 50 eligible respondents. We further purposively sampled two additional woredas from Oromia and SNNPR (Bunno Bedelle and Chencha, respectively) where other evaluative work was also taking place, to triangulate findings in other evaluation components.

For each of the seven QI programme woredas chosen for data collection, we chose one matched woreda from the same region which was not subject to QI activities. Repeated cross-sectional data were available, however, on maternal health care utilization from DHS data sets. DHS data report at the regional level, so we used GPS co-ordinates from the DHS data to identify woreda and estimate the woreda-level health service utilization levels. Using the three most recent DHS surveys (2016, 2011 and 2005), we estimated woreda-level means of the three DHS variables relating to a woman’s most recent birth: the proportion of women delivering in a health facility, the proportion who received no prenatal care and the proportion who received no postnatal care. We used a simple and transparent approach to this, selecting the comparison woreda in each region with the closest utilization compared to QI woreda across three equally weighted indicators.

#### Survey implementation

In a cadre-stratified sample, we sought to interview 50 participants per woreda. We interviewed around four maternal and child health care providers from the hospitals and two from each health centre, around five HEWs from each health centre and one HEW from each health post. We also interviewed non-patient-facing staff: the heads or clinical directors of the woreda, each hospital and each health centre. We did not specifically include or exclude participants who were part of QI collaboratives or who had attended learning sessions.

We obtained permission letters from woreda health offices and sampled providers from all health facilities in the woreda. Woreda health offices were also asked to report any other QI programmes or initiatives in the area, and no major activities were identified outside of the programme under evaluation. In each health facility we obtained a list of all maternal and newborn health (MNH) providers and randomly selected participants for interview. Their names were written in alphabetical order next to a column of randomly generated numbers and interviewers sequentially chose participants from the smallest random number upwards until the requisite number of participants was reached. If participants were not available, we sought to arrange interviews via phone and returned to the facility up to three times before classifying them as unreachable and selecting the next worker from the list.

Quantitative data collection was conducted by seven research assistants who received one-week training at the start of the data collection process. Each worked in regions they were familiar with to assist with community entry and mitigate language issues. In both survey rounds, tools were translated to Amharic and Oromiffa languages and data were entered on tablet computers using Open Data Kit software (www.opendatakit.org) and analysed in STATA.

In the endline survey, conducted 15 months after the baseline survey and immediately after the final learning session of the QI programme, we sought to re-interview all participants regardless of whether they were in the same facility as at baseline. We used mobile phone numbers provided at baseline and alongside information from woreda health office staff to locate participants. If participants could not be located after three attempts via phone and other channels, they were deemed to be uncontactable. When all participants had been either contacted or deemed uncontactable, we re-sampled at the facility level using the same sampling methods as at baseline.

#### Qualitative data collection

We conducted 22 qualitative in-depth interviews in the Oromia region, chosen due to proximity to the study team in Addis Ababa, around three-quarters of the way through QI programme implementation in April and May 2019, 13 in a QI woreda and nine in a comparison woreda. Woredas were chosen due to good pre-existing links with researchers and proximity to Addis Ababa. Initially 15 interviews were planned, however an additional seven were added after preliminary analysis of transcripts identified that additional data were needed to achieve saturation.

In the chosen woreda, interviewers contacted participants from the first round of quantitative data collection. Interviewers called participants chosen from a cadre-stratified random sample of baseline quantitative study identification numbers. Topic guides were piloted with the study population and included reasons for choosing their profession, motivating and demotivating factors, and—in QI woreda only—the influence of QI programme. Two interviewers with qualitative experience received two-day training on study aims, topic guides and ethical issues including informed consent. Training included how to probe and reduce desirability biases in responses. Both interviewers were Ethiopian women under 35 years of age. Interviews were conducted in Amharic or Oromiffa, directly transcribing and translating transcripts into English for analysis.

Data collection and analysis followed a sequential mixed-methods approach. Baseline quantitative data were collected in April and May 2018 immediately prior to the start of the QI programme’s test of scale phase. Qualitative data were collected in April and May 2019. Endline quantitative data were collected in June 2019 at the end of the programme’s test of scale phase.

#### Data analysis

Our main analysis is within cadres, where we split the sample into patient-facing midlevel care providers and HEWs, and non-patient-facing staff.

#### Change in health worker knowledge

To analyse changes in health worker knowledge, we counted the number of appropriate responses each respondent gave to each of the six stages of the clinical vignette and inserted them as the outcome in Equation (1). We also used item response theory to analyse response data by weighting each knowledge component by a latent measure representing the difficulty of each (Das and Hammer, 2005).

#### Factor analysis of health worker motivation

To understand the dimensionality of the motivation measure at baseline we conducted an exploratory factor analysis. Factor analysis uses the covariance between variables to identify distinct underlying groups of variables which are correlated with one another. First we dropped variables from the list of 30 questions which had poor psychometric performance, defined by having (1) more than 10% missing data, (2) being given the same score by over 80% of participants and (3) with factor loadings less than 0.4 (Ferguson and Cox, 1993; Tabachnick *et al.*, 2007). As recommended in the literature, we used a threshold of 0.4 to reflect a strong relationship with a factor, and the optimal number of factors was established through a scree test and multiple runs (Chandler *et al.*, 2009). We used maximum likelihood ProMax oblique rotation to reduce the number of variables with high loadings and to allow factors to be correlated and assumed that construct validity was indicated by loading at least three variables per factor and absence of substantive cross-loading (Costello and Osborne, 2005). We ran models with between two and five factors, removing variables which did not load on any factor to 0.4, and used eigenvalues >1 as selection criterion alongside identifying models with substantial cross-loading of variables to factors. Mean scores for each factor were calculated by summing the responses for each factor by respondent and dividing by the number of variables. We assigned qualitative titles to factors based on the variables categorized into each.

#### Change in health worker motivation

In quantitative motivation analyses, we used individual variables and the factors identified in baseline data to explore variation in motivation over time and across geographic areas. We take an intention-to-treat approach, analysing data from all respondents regardless of their participation in QI activities. First, for each cadre we report the mean scores on each variable and for each factor at baseline and endline, and in QI and comparison areas. Second, we ran linear ordinary least squares difference-in-difference regression models to explore if there were differences in individual variables and mean factor scores for each motivation dimension between QI and comparison areas.

(1) \begin{align*}{Outcom}{e_i} &= {\beta _1}{QI}\_{are}{{a}_i} + {\beta _2}{endlin}{{e}_i} \nonumber \\ &\quad+ {\beta _3}\left( {{Q}{I}\_{area}_i}*{endlin}{{e}_i} \right) + {\varepsilon _i}\end{align*}

where $Outcom{e_i}$ is a motivation or knowledge variable or mean factor Likert score for an individual $i$, $QI\_area$ a binary variable denoting whether the woreda of individual $i$ received the QI intervention and $endlin{e_i} $a binary variable denoting whether data were collected in baseline or endline data surveys. Regression models were specified with robust standard errors clustered at the woreda level with health facility fixed effects.

#### Analysis of qualitative data

As the purpose of the qualitative work was to triangulate facets of motivation and understand mechanisms of change, we first developed a coding framework based on emergent patterns in the exploratory factor analysis. We added one additional code to capture perspectives on the impact of the QI programme on motivation. Although we were prepared to adapt this coding framework in light of emergent themes from the data which did not fit into it, no changes were deemed necessary to the initial coding framework developed from baseline quantitative data. After the coding frame was developed, the lead author wrote narrative summaries of relevant themes and subthemes and identified relevant quotes.

#### Ethical considerations

The research protocol and tools were reviewed and approved by the Observational/Interventions Research Ethics Committee and the Ethical Review Committee of the authors’ institutes.

## Results

### Characteristics of quantitative sample

Table 2 shows the characteristics of the quantitative sample for each cadre at baseline and endline. HEWs comprised the largest group sampled (209 at baseline and 202 at endline), followed by midlevel care providers (148 at baseline and 166 at endline) and non-patient-facing staff (36 at baseline and 40 at endline). Mean motivation, as assessed by simply asking respondents to describe their motivation in their current role, was 2.2 (on a scale from excellent = 1, very good = 2, good = 3, fair = 4 and poor = 5) at baseline and endline, and did not vary by QI and comparison areas. As expected, HEWs were an almost exclusively female cadre whilst non-patient-facing staff were 96% male. The HEW sample at endline was younger (26.8 years) than at baseline (27.6 years, difference 0.85 years, *P* = 0.05). On average compared to other cadres, HEWs had worked in their current facility the longest (40 months) and non-patient-facing staff had worked in the health system the longest (45 months). Reported annual salaries varied across cadres with the managerial cadre of non-patient-facing staff earning the most (Ethiopian Birr, ETB 6053), followed by midlevel care providers (ETB 4191) and HEWs (ETB 3290). The non-patient-facing cadre was the most likely to report unauthorized absenteeism in the previous 3 months where 33% reported any absenteeism, although we cannot rule out equality in absenteeism across cadres.

**Table 2.** Characteristics of quantitative sample

	**Midlevel care providers**			**Health extension workers**			**Non-patient-facing staff**		
	**Baseline**	**Endline**		**Baseline**	**Endline**		**Baseline**	**Endline**	
	**(*n* = 148)**	**(*n* = 166)**	***P*-value**	**(*n* = 209)**	**(*n* = 202)**	***P*-value**	**(*n* = 36)**	**(*n* = 40)**	***P*-value**
Age (years)	27	29	0.06	27	28^a^	0.35	31^c^	33^b^^,^^c^	0.07
Female	0.52	0.51	0.88	1^a^	1^a^	0.09	0.06^b^^,^^c^	0.03^b^^,^^c^	0.50
Time at current facility (months)	29	31	0.25	38^a^	41^a^	0.04	21^b^^,^^c^	30^b^	0.05
Time working in health system (months)	36	39	0.07	40^a^	43^a^	0.02	434^c^	46^c^	0.18
Gross salary (ETB, monthly)	4191	4503	0.11	3290^a^	3443^a^	0.09	6053^b^^,^^c^	6056^b^^,^^c^	1.00
Poor well-being	0.66	0.64	0.84	0.7	0.77^a^	0.12	0.67	0.6^b^	0.55
Unauthorized absenteeism in previous 3 months (%)	19	20	0.73	25	24	0.80	33	33	0.94
Overall motivation [mean, scale excellent (=1) to poor (=5)]	2.3	2.2	0.25	2.2	2.2	0.68	1.8^c^	1.9	0.60
Knowledge: ‘History—Previous pregnancies’ (number of correct items reported/11)	4.6	5.4	<0.01	3.9^a^	4.4^a^	<0.01			
Knowledge: ‘History—Current pregnancy’ (number of correct items reported/14)	5.6	6.2	0.03	4.7^a^	4.6^a^	0.53			
Knowledge: ‘History—medical history’ (number of correct items reported/12)	3.6	4.7	<0.01	2.6^a^	3.2^a^	0.00			
Knowledge: ‘History—Examinations’ (number of correct items reported/11)	6.1	6.6	0.11	4.8^a^	4.5^a^	0.11			
Knowledge: ‘History—Investigations’ (number of correct items reported/15)	6.0	6.9	<0.01	3.6^a^	4.1^a^	0.01			
Knowledge: ‘History—Drugs supplied’ (number of correct items reported/5)	1.1	1.8	<0.01	1.1	1.7	<0.01			
QI: Aware of QI programme^d^ (%)		86			72^a^			94^b^	
QI: Member of QI team^d^ (% of those aware of QI programme)		69			37^a^			88^b^	
QI: Number of QI meetings and learning sessions attended^d^ (% among members of QI team)		10			9			9	

a Strong evidence of difference between patient-facing midlevel care providers and HEWs (*P* < 0.05), at each time point.

b Strong evidence of difference between HEWs and non-patient-facing staff (*P* < 0.05), at each time point.

c Strong evidence of difference between patient-facing midlevel care providers and non-patient-facing staff (*P* < 0.05), at each time point.

d At endline, among participants in QI woreda.

Finally, 79% of the sample in QI areas were aware of the QI programme at endline, and there was no evidence of differences in awareness of the QI programme across cadres. Of those aware of the QI programme (*n* = 164), just 37% of HEWs were members of a QI team, compared with 70% of midlevel care providers and 88% of non-patient-facing staff. Of those in a QI team (*n* = 136), midlevel care providers and non-patient-facing staff attended 10 QI meetings and learning sessions on average, HEWs attended nine.

### Health worker motivation

#### Dimensionality of motivation

No variables met our pre-specified criteria for poor psychometric performance, so all were included in analysis. The three-factor model shown in Table 4 was deemed to fit the data best, and after analysing their constituent variables we gave each a qualitative title. Factor 1 was described as ‘Helping others and reaching personal goals’, Factor 2 as ‘Pride and self-efficacy in job’ and Factor 3 as ‘External recognition and support (financial and managerial)’. The scree plot from the factor analysis is shown in Supplementary Figure S1. Supplementary Figure S2 shows the distribution of Likert scale responses across indicators. In the following section we present the deductive analysis of qualitative data in the framework which arose from the quantitative factor analysis, to triangulate quantitative findings and elucidate on the construction of motivation.

**Table 4.** Exploratory factor analysis

**Variable**	**Factor 1**	**Factor 2**	**Factor 3**
My work is important because I help people	0.65	0.12	0.35
I am respected in my community for the work I do	0.58	0.36	0.14
I am keenly aware of the career goals I have set for myself	0.57	0.30	0.28
I feel committed to my role	0.56	0.45	0.21
If I do well at work, I will achieve my goals	0.54	0.32	0.21
I am willing to do more than is asked of me in my role	0.47	0.29	0.13
I can solve most problems I have at work if I work hard	0.37	0.29	0.29
I can complete all of the work I am expected to do	0.38	0.41	0.06
I feel like performing the duties required of me	0.35	0.53	0.22
I am proud of the work I do	0.33	0.57	0.25
I am proud to be working in my role	0.33	0.65	0.18
In general, I am satisfied with my role	0.14	0.57	0.12
My job makes me feel good about myself	0.14	0.54	0.36
It is important that I do a good job so that the health system works well	0.48	0.19	0.48
Training sessions that I attend are worthwhile and add benefit to my career path	0.37	0.15	0.49
To be motivating, hard work must be rewarded with more status and money	0.31	0.06	0.58
I am strongly motivated by the recognition I get from other people	0.26	0.34	0.44
I gain knowledge from being in this role	0.18	0.44	0.62
My supervisors and managers are supportive of me	0.14	0.36	0.36

Notes: Variables shown are those which loaded >0.4 onto at least one factor. Where variables loaded above 0.4 on more than one factor, they are shown in the factor in which they loaded the highest. These factors are:

Factor 1—Helping others and reaching personal goals.

Factor 2—Pride and self-efficacy in job.

Factor 3—External recognition and support (financial and managerial).

##### Factor 1: helping others and reaching personal goals.

The opportunity to help others was seen as a key motivator across all three cadres. Health providers and HEWs frequently mentioned receiving job satisfaction from helping mothers and new-borns:

*As a midwife the work I do and the payment I receive do not match. The salary is not sufficient but when I see mothers deliver safely and when I see the new-borns, I become very satisfied an* *d I consider that as my salary.* [Midwife, 28 year old female, QI area]

*I like my work in general because it makes me do tangible work to save life of people and keep their health. I feel very happy when patients come to me; I treat them and I see them get their health back* [HEW, 25 year old female, comparison area]

Non-patient-facing staff also noted the importance of helping others—both the staff they manage and the patients in the facilities they run:

*When I see HEWs under my supportive supervision win, I feel happy, as it is fruit of my* *work. We win most of the time.* [HEW supervisor, 31 year old male, QI area]

Participants also mentioned the opportunity to participate in training, in the QI project and more broadly gain professional qualifications as motivating factors.

##### Factor 2: pride and self-efficacy in job.

Highly skilled care providers and HEWs identified the lack of equipment as a key demotivating factor, as this inhibited their ability to provide an effective service to mothers and new-borns.

*Because of lack of supply, absence of quality care, or manpower, if patients are referred to another hospital I feel sad. Most of the time we refer patients because of absence of supplies; I am there to help them but I can’t do anything so I feel sad* [medical doctor, 32 years old, QI area]

In addition to physical equipment, providers saw training as a way to improve their effectiveness in their role, using skills gained to provide better services, which in turn improved their motivation:

*I like when capacity and confidence I get from trainings help me solve tangible problem. For example, with training I received on nutrition, I feel glad when I help children and see them improved in 15 days or so. [* *…] Before the training, I had no capacity to provide the care. Therefore, I like when the training I received enable me provide care that I couldn’t provide before.* [HEW supervisor, 31 year old male, QI area]

##### Factor 3: external recognition and support (financial and managerial).

Perceived low salaries were identified by nearly all participants across cadres as a key demotivating factor, and among non-patient-facing staff as demotivating themselves and the patient-facing staff in their teams

*As HEW the salary is not good. As I said before the work of HEW is very difficult so the salary is unsatisfactory when I compare with our hard work. We are serving our community so this makes us to work not the salary.* [HEW, 30 year old female, comparison area]

*The salary of health professionals is less than daily labourers so the least paid are health professionals. This is one factor that makes me to dislike my job* [Nurse, 54 year old male, QI area]

The availability of work in the private sector for midlevel care providers was identified as a factor which demotivated participants to work in the public sector, although these opportunities were not available to HEWs.

*When the salary is less, workers have no motivation for work. Since their salary is low they always think about working in private health facilities rather than focusing on their work at the health centre. This includes myself and workers under me. No one has interest in staying in government health facilities.* [Head of health centre, 30 year old male, QI area]

#### Effect of QI on health worker knowledge

We found some evidence of greater increases in antenatal care knowledge in QI areas than in comparison areas among HEWs, and weak evidence of increases among midlevel care providers. Table 3 shows the six dimensions of the vignette where the dependent variable for each column is the number of correct responses a participant mentioned when asked an open-ended question—coefficients are interpreted as the change in the number of correct items listed by respondents in QI compared to comparison areas. For example, the number of correct history items for previous pregnancies listed by respondents out of the list of 11 correct responses increased by 0.72 more on average in QI than comparison areas. QI areas saw an increase in correct history items asked about current pregnancy (1.29 items more on average), examinations (0.79 items more), and among HEWs for ordering drugs and supplies (0.5 items more on average). Knowledge increased more among midlevel care providers in areas of history taking around previous pregnancies and examinations, whilst increases were greater among HEWs in history taking around a current pregnancy and ordering correct drugs or supplies, knowledge of the latter did not increase among midlevel care providers. Column 7 of Table 3 sets the dependent variable as the total of correct responses given across all domains to give an overall assessment of knowledge change; we find that overall knowledge among HEWs increased in QI areas more than comparison areas, although the same pattern was not observed among midlevel care providers. Results from analyses using item response theory (Supplementary Table S1) are consistent with the above findings, except for history taking around current pregnancy which switches sign, indicating that providers mentioned less-common knowledge items fewer times in QI areas. Finally, we conducted a subgroup analysis and found no evidence to suggest knowledge changed differentially among members of QI teams compared with non-members in intervention woreda.

**Table 3.** Difference-in-difference analysis of knowledge changes by cadre

	**(1)**		**(2)**		**(3)**		**(4)**		**(5)**		**(6)**	
	**History: Previous pregnancies**		**History: Current pregnancy**		**History: Medical history**		**Examinations**		**Investigations**		**Drugs/** **supplies**	
	**Coeff.**	**CI**	**Coeff.**	**CI**	**Coeff.**	**CI**	**Coeff.**	**CI**	**Coeff.**	**CI**	**Coeff.**	**CI**
*Panel A: Midlevel care providers*												
Difference-in-difference coefficient (QI*Endline)	1.03**	(0.4–2.01)	0.91	(−0.38–2.2)	0.13	(−0.71–0.97)	1.15*	(−0.05–2.34)	0.47	(0–0.52-1.47)	0.22	(−0.15–0.60)
Observations	307		307		307		307		308		307	
R-squared	0.066		0.031		0.091		0.032		0.044		0.244	
*Panel B: HEWs*												
Difference-in-difference coefficient (QI*Endline)	0.57*	(−0.05–1.19)	1.66***	(0.8–2.51)	0.35	(−1.67–0.87)	0.68*	(−0.11–1.46)	0.64	(−0.14–1.42)	0.50***	(0.28–0.73)
Observations	401		401		396		399		375		399	
R-squared	0.045		0.038		0.067		0.016		0.026		0.305	

Notes: Coefficients are interpreted as the change in the number of correct items listed by respondents in QI compared with comparison areas. Each column shows the results of an ordinary least squares regression model where the dependent variable is the number of relevant items that a patient-facing health worker mentioned when asked an open question regarding the care of Mrs X, a married woman of 26 years who has moved into the area and comes to see you for the first time. The dependent variable of Column 1 is the number of items respondents report from a list when asked ‘what questions would you ask Mrs X about her previous pregnancies’. In Column 2, the question asks about her current pregnancy. In Column 3, the question asks about her medical history. In Column 4, the question asks what physical examinations would be performed. In Column 5, the question asks what investigations would be performed. In Column 6, the question asks what drugs or supplies the respondent would provide to Mrs X. Standard errors are clustered at woreda level, regressions include facility fixed effects.

#### Effect of QI on motivation

**Figure 2.**
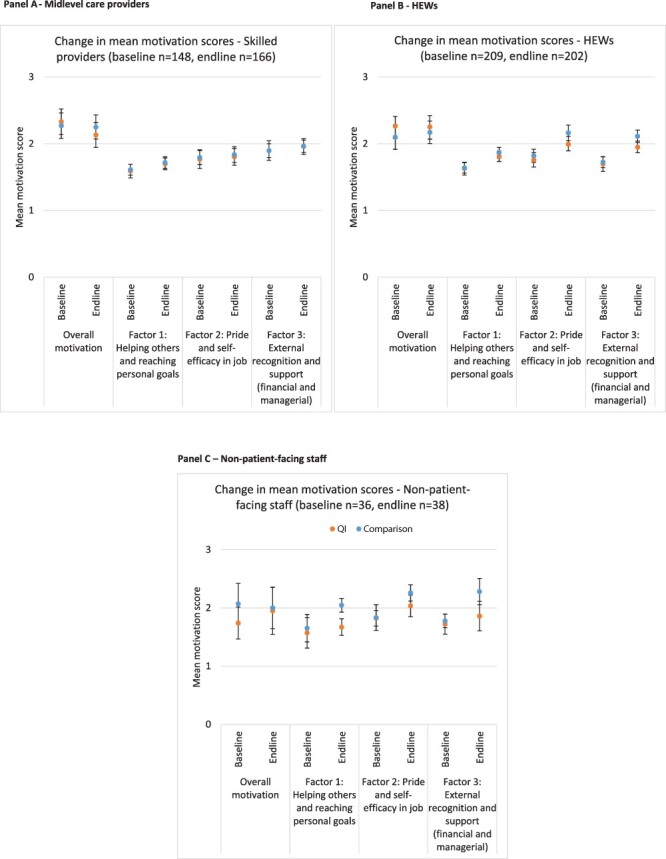
Change in mean factor scores at baseline and endline, by QI/comparison area in each cadre. Panel A—Midlevel care providers. Panel B—HEWs. Panel C—Non-patient-facing staff. Notes: In each panel, the vertical axis shows the mean motivational score. Statistically meaningful motivational scores between baseline and endline are indicated on the horizontal axis, where *** denotes *P*-value < 0.01. Overall motivation refers to the question: ‘How would you rate your overall motivation at your current work?’.

Quantitative analyses show some evidence of increased motivation in both QI and comparison areas. Figure 2 shows motivation measures at baseline and endline in QI and comparison areas, by cadre. Supplementary Figure S4 shows this for the pooled sample, and Supplementary Table S2 regression models show the same thing. Among midlevel care providers and non-patient-facing staff in QI areas, there is no evidence that motivation changed between baseline and endline. However, among non-patient-facing staff in comparison areas, there is strong evidence that motivation increased between baseline and endline. Among HEWs, there is strong evidence that motivation increased in both QI and comparison areas between baseline and endline; however, this increase in motivation was greater in comparison areas than QI areas. Supplementary Figure S3 shows analyses conducted on the pooled sample of all three cadres and demonstrates strong evidence of motivation increases in both QI comparison and comparison areas—this is primarily driven by the increases observed among HEWs, who comprise over half of the sample. Finally, we conducted a subgroup analysis and found no evidence to suggest motivation changed differentially among members of QI teams compared with non-members in intervention woreda.

Contrary to the quantitative findings, qualitative data from QI areas implied that participants, of all cadres, who had been directly involved in QI felt that QI activities were beneficial to health worker motivation, across all three factors. Even when directly prompted to identify negative aspects of the QI programme on motivation, very few participants were able to. When asked to describe positive elements of the QI programme on motivation, midlevel care providers and HEWs repeatedly spoke of how training sessions left them feeling confident and able to provide good quality care, which they in turn found highly motivating. For example, the following was jointly coded as both ‘Helping others and reaching personal goals and Pride and self-efficacy in job’:

*[The QI programme has had a] positive effect on motivation of workers. People, by nature, get motivated when they get good outcome out of the things they do. And through QI program workers improved their service quality which made them to be happy by the work they do. And this further motivated them to continue providing quality care*. [Health officer, 30 year old male, QI area]

Several participants mentioned being motivated by the longer-term follow-up if the QI programme compared with other training interventions, which aligned with ‘External recognition and support’, e.g.:

*IHI is what makes us motivated. Some organizations after they give training they disappear. No follow up and supervision at all. But IHI staff come for supervision every three months so this increases staff motivation. Their follow up makes us motivated.* [Midwife, 28 year old female, QI area]

The ‘External recognition and support’ theme also captured that one of the few negative aspects identified was that not all staff were able to participate in the QI programme. Participants noted that that colleagues who did not attend were not so invested in changes made due to the QI programme compared to colleagues who attended learning sessions.

*They cascade [information from QI sessions] [* *…] But only the one who took the training works with motivation. And, if you ask the one who did not take the training to work he may not work being motivated as the one who took the training.* [Care provider, 29 year old male, QI area]

We did not detect demotivating factors among those who were in QI areas but not part of QI teams, and some participants noted that the perceived improvement in patient care and skills for those part of the QI team was motivating for them:

*The quality improvement program in our woreda is good [* *…] This program improves the quality of my colleagues work so this motivates me to work with them*. [Care provider, 54 year old male, QI area]

There were no substantive differences in the types and frequency of mention of non-QI themes in qualitative data between QI and comparison areas. In general, qualitative data suggested that the QI programme had positive impacts on health worker motivation. In all QI area interviews, interviewers probed respondents to identify and discuss potentially negative aspects of the QI programme but almost all respondents were not able or willing to do so. Quantitative data triangulate these positive effects of QI on motivation, as motivation increased in all cadres between baseline and endline. However, qualitative data are not able to explain quantitative findings that motivation increased more in comparison areas than QI areas.

## Discussion

This study measured changes in the motivation and knowledge of three cadres of health workers in the context of a QI programme in Ethiopia. We found strong evidence that health worker knowledge increased more in QI areas than comparison areas, particularly among HEWs. We did not find evidence that motivation changed in QI areas relative to comparison areas, in any cadre. Qualitative data suggested that the QI programme had unambiguously positive effects on health worker motivation across all cadres who had been directly involved in the QI initiative; however, quantitative analyses do not triangulate this finding. In QI areas, motivation improved only among HEWs, whilst motivation in comparison areas increased among HEWs and non-patient-facing staff. In a pooled analysis, we estimated that motivation increased more in comparison areas than QI areas.

On average, overall motivation was reported as good or very good by respondents, with HEWs reporting higher motivation than midlevel care providers. An exploratory factor analysis identified three discrete factors to categorize variables: (1) helping others and reaching personal goals; (2) pride and self-efficacy in job and (3) external recognition and support (financial and managerial). Qualitative data triangulated this definition of motivation construction, highlighting specific motivators and demotivators within each.

Because we measured motivation and knowledge in many woredas in Ethiopia and included three cadres of health workers—including HEWs whose motivation has not previously been comprehensively assessed—this study may provide a comprehensive picture of motivation across cadres in Ethiopia. In addition, no previous work has sought to measure how QI programmes affect health worker motivation in any setting, nor explore concurrent knowledge changes. In particular, findings of increased knowledge are consistent across patient-facing cadres of midlevel care providers and HEWs; similarly, the lack of evidence for changes in motivation is also consistent. We note that the main demotivating factors identified in qualitative data were things which the QI programme did not address, such as health worker salaries or a lack of equipment. Although the QI programme had the intention to fill the equipment gap of the health facilities, the plan was not fully successful and a subset of facilities received the equipment at or after the completion of the programme and others received only minor supplies during the programme.

The inconsistency between motivation and knowledge findings in this study can perhaps be attributed to the fact that the QI intervention we evaluate is complex and multifaceted, whilst motivation and knowledge—although both are positively related to quality of care—may act in different directions in response to a QI intervention, or over different periods of time. For example, an increase in workload needed to increase knowledge may temporarily demotivate health workers before motivation may be later increased through self-efficacy—the latter was more salient in our qualitative data than the former. A priori, we were not able to identify a clear hypothesis of whether motivation and knowledge would move in the same direction—represented by a dashed two-way arrow in the hypothesized theory of change. This relationship remains difficult to conceptualize and would merit greater exploration in the future to understand how QI programmes can maximize impact.

This study is consistent with findings in the literature that motivation is generally high among health workers in Ethiopia, but that the determinants of motivation are difficult to elicit (Dagne *et al.*, 2015; Weldegebriel *et al.*, 2016; Selamu *et al.*, 2017). Although it is difficult to compare the absolute level of motivation across studies, the mean score at baseline among participants was 2.2—between ‘2=good’ and ‘1=very good’—consistent with other studies reporting moderate to high motivation among health worker is Ethiopia (Weldegebriel *et al.*, 2016; Tesfaye, 2017). The construction of motivation we identified was similar to that of other studies, which have identified motivators at the individual (intrinsic and extrinsic), community and organizational levels (Weldegebriel *et al.*, 2016; Tesfaye, 2017). Qualitative data in particular reinforced findings from other studies that health workers are particularly motivated by the opportunity to improve the health of their patients (Serneels *et al.*, 2010) and perceived managerial quality (Weldegebriel *et al.*, 2016). As identified elsewhere (Lindelow and Serneels, 2006), low salaries were repeatedly mentioned in qualitative data as causing absenteeism, specifically to neglect public roles for more lucrative work in the private sector. Importantly, although we did not observe substantive changes to motivation, we find strong evidence that the QI programme increased health worker knowledge in key domains; this is aligned with other studies which observed small positive impacts from QI programmes on health worker practices (Larson *et al.*, 2020).

However, this study has important limitations. The quantitative motivation tool was adapted from studies conducted in other countries, and although these were sub-Saharan African settings, Ethiopia has a distinct cultural, political and health system which may have affected the sensitivity and internal or external validity of the quantitative tool. In addition, the qualitative tool was adapted from a community health worker study in Uganda, and although adaptations were made to make it relevant across cadres, some important areas may have been omitted. Motivation scores were generally high at baseline, which potentially led to ceiling effects whereby we are not able to detect small changes to already-high reported motivation. Although none were reported when study staff asked woreda leadership, other programmes in QI or comparison areas may have sought to improve motivation or knowledge. Qualitative interview guides were based on the analysis of baseline quantitative data, which may have led to the omission of important topics from interviews. In addition, qualitative data were collected at one time point in the last month of the QI programme and this may have been after demotivating pressures had reduced. There may not have been sufficient power at the cadre level to detect changes in motivation, particularly in the presence of heterogeneity in QI areas arising from some participants not being involved in the QI programme or being part of QI teams Comparison woreda were selected based on maternal healthcare utilization data because these were widely available from DHS surveys, however, may not have produced a reliable comparison group for health worker motivation. Although a criticism of all health worker motivation studies, quantitative data were self-reported and may be subject to acceptability biases. QI data have been found previously to be influenced by whether questions were framed positively or negatively (Ballard, 2019), yet although such biases would overestimate cross-sectional estimates of motivation, these would likely cancel out in our analyses over time or by QI or comparison area, assuming biases were not affected by the programme. Additionally, data were not available on potentially important contextual factors which may have enhanced or inhibited the impact of the QI intervention, e.g. facility readiness or local leadership. Finally, without access to data on health worker performance or objective measures of the quality of care provided, we are not able to explicitly explore the association between health worker motivation and health outcomes, although clinical vignettes have been shown to correlate well with other quality of care measures (Peabody *et al.*, 2004). Future research could seek to understand the relationship between motivation and knowledge and how this changes over time and in response to policy interventions.

## Conclusion

We used a mixed-methods approach to investigate whether health worker knowledge and motivation changed as a result of the ‘Ethiopia Health Care Quality Initiative’. We found some evidence that clinical knowledge improved more among patient-facing staff in QI areas than comparison areas, but little evidence that the programme impacted motivation, with some divergent findings between qualitative and quantitative data. Although motivation and knowledge are not necessarily primary goals of QI programmes, they can be important antecedents to good quality care, and it is critical that QI programmes measure possible intended and unintended consequences among health workers.

## Supplementary Material

czab094_Supp

## Data Availability

The data sets used and/or analysed during the current study are available from London School of Hygiene and Tropical Medicine Data Compass.
