# Supplementary material for: Changes in health worker knowledge and motivation in the context of a quality improvement programme in Ethiopia
Source: Health Policy Plan. 2021 Aug 10;36(10):1508–20. doi: 10.1093/heapol/czab094 (PMC8597962; doi:10.1093/heapol/czab094)
Supplement: czab094_Supp [file czab094_supp.zip › HPP Supp material_revised-clean 11.6.docx]

Online supplementary material for

Changes in health worker knowledge and motivation in the context of a quality improvement programme in Ethiopia

# Supplementary file 1: Additional information on the QI programme

**The following information is taken from the following source: The Ethiopia Health Care Quality Initiative: Design and Initial Lessons Learned, Institute for Healthcare Improvement, 2020**

**Figure S1:1 Collaborative Design**

**Learning Session 3**

**Learning Session 2**

**On-site coaching to support teams to improve system and clinical skills gaps**

**12-18 months**

**Learning Session 1**

**Address gaps in clinical and QI skills and supplies**

**Finalize change package, publicize & spread**

**Learning Session 4**

**Action Period 2**

**Action Period 3**

**Action Period 1**

**Identify focus area and core indicators**

**Conduct Baseline Assessment**

**QI training for Facility Leaders**

Launch Activities

At the initiation of each collaborative, woreda health officers and facility-level leadership were trained in QI processes, after which they conducted a baseline facility assessment to identify the gaps in MNH care delivery. From this assessment, action plans were developed with health facility managers to address core input gaps, including provision of clinical trainings in emergency obstetric and neonatal care. IHI also supported facilities to mobilize resources to close structural gaps such as water and electricity, mainly through data-based advocacy. Targeted financial support was provided by IHI when local resources were exhausted.

*Learning Sessions and Coaching*

Each prototype collaborative was about 15 months, with four 2-3 day learning sessions separated by three action periods. Learning session participants received financial support at local rates to cover transportation, accommodation and meals. In the first learning session, participants were trained in QI methodologies and the collaborative model. After identifying the key health system drivers of better performance using driver diagrams, and defining the core improvement collaborative indicators that would be the target of improvement, participants used their own facility baseline data to identify key MNH quality gaps. From this, aim statements were constructed and change ideas developed to achieve those aims. These ideas were tested and refined during action periods using Plan-Do-Study-Act (PDSA) cycles, with support provided by IHI project officers and woreda-level coaches during QI coaching visits. Change ideas that were determined to be effective, based on run chart rules and qualitative feedback from QI teams, were included in a final change package.

Successes and challenges in implementing change ideas and clinical bundles were shared across teams during learning sessions. Patient-centered care, including respectful maternity care, defined as care that is “humane and dignified, and delivered with respect for women's fundamental rights”, was a core emphasis of learning sessions and QI projects. After building will for community engagement amongst health care workers, community members also attended the last learning session in the prototype phase, providing feedback on their priorities and experience of care, and the proposed change package. We combined clinical mentorship with QI coaching during action periods; coaches observed clinical processes and supported patient care as needed, and provided QI project coaching with data review. This integrated approach provided real-time clinical support and helped QI coaches understand facility-level systems better. By reviewing data together and comparing reported data to source registers, coaches also addressed data quality through the coaching process.

Figure S1:2, taken from the Ethiopian Health Care Quality Initiative Project Overview, demonstrates the mechanisms through which the programme was hypothesised to effect change. The relevant mechanisms that this study seeks to evaluate are highlighted. Specifically, “Improved quality of care at health institutions” was sought through increasing the “availability of care and respectful health personnel”, providing training, mentorship and seeking to improve health worker efficiency.

Table S1:1 highlights how these different programme activities were hypothesised to impact either knowledge or motivation among health system workers. However, we note that the QI programme is a complex intervention, and it is not possible to isolate the specific influence of each aspect of the QI programme on motivation or knowledge.

| **Activity** | **Domain of hypothesised impact among health workers** |
| --- | --- |
| Training in key MNH national protocols | - Motivation – hypothesised to increase through feeling that skills are valued and worth developing, individual/facility being chosen as part of QI programme - Knowledge – hypothesised to increase through exposure to training programmes and materials |
| Onsite mentorship to maintain skills and address skills gaps | - Motivation – hypothesised that exposure to mentors will increase sense of worth and purpose in job role, and feel that individual is worth developing - Knowledge – hypothesised to increase through exposure to mentors with greater knowledge |
| Maximize efficiency of existing facility staff | - Motivation – could increase or decrease motivation. If efficiency is maximised at expense of staff wellbeing or through placing greater pressure on staff time, motivation may decrease. If efficiency is increased to allow staff greater opportunity to effect change and increase impact, motivation may increase |
| Professionals get regular updates on the management and prevention of key causes of mortality | - Motivation – hypothesised to increase motivation through highlighting link between staff actions and ultimate wellbeing of patients - Knowledge – hypothesised to increase through information sharing, and discussion of factors contributing to changes |

**Table S1:1: Relationship between QI programme activities and hypothesised relationship between health worker motivation and/or knowledge**


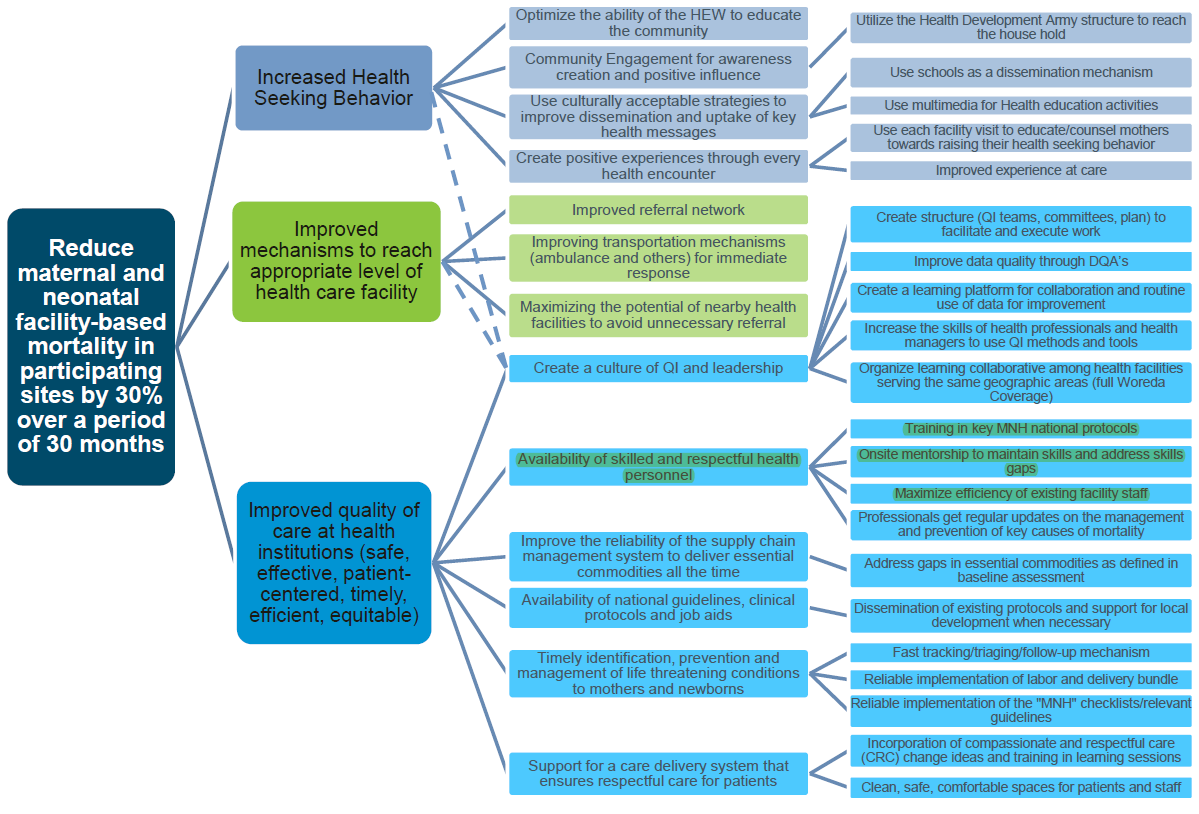


**Figure S1:2: Diagram showing how MNH collaboratives were hypothesised to lead to improved health outcomes through QI programme activities. Highlight formatting added by authors of this study, to demonstrate the areas and activities evaluated here.**

# Supplementary file 2: Health worker knowledge tool

**Question 1:**

Mrs. X, a married woman of 26, has recently moved into the area and comes to see you for the first time. She is obviously pregnant and reports that she has not received any antenatal care for this pregnancy. Please tell me what questions you would ask Mrs X and what actions you would take.

What questions would you ask Mrs X about her previous pregnancies?

DO NOT READ OPTIONS ALOUD. FOR EACH OPTION, NOTE WHETHER THE HEALTH WORKER MENTIONED THIS ACTION OR NOT.

**Q1 responses (select many):**

| Number of previous pregnancies |
| --- |
| Number of live births |
| Number of miscarriages/stillbirths/abortions |
| Any bleeding during last pregnancy |
| Mode of previous deliveries |
| Birth weight of previous babies |
| Tetanus immunization during last pregnancy |
| Any genetic abnormalities in previous babies |
| Place of previous deliveries |
| Year(s) of previous deliveries |
| History of twin birth |

**Question 2:**

What questions would you ask Mrs X about her current pregnancy?

**Q2 responses (select many):**

| Last normal menstrual date |
| --- |
| Any health problems now |
| Any contractions |
| Any vaginal bleeding |
| Blurring of vision |
| Edema |
| Leakage of liquid |
| Decreased or absent foetal movement |
| Quicknening |
| Weight loss/gain |
| Any nausea or vomiting |
| Are you taking any medication? |
| Have you had tetanus immunization? |
| If the pregnancy was planned or unplanned |

**Question 3:**

What questions would you ask Mrs X about her medical history?

**Q3 responses (select many):**

| Any history of high blood pressure |
| --- |
| Any history of diabetes |
| Any previous STI/HIV? |
| Previous use of family planning? |
| Ever had a pap smear? |
| History of heart disease, malaria, liver disease or goiter |
| Family history of hereditary disease |
| Any allergies to medication |
| Current or previous smoker |
| Any history of alcohol use |
| Any history of illicit drug use |
| Any history of epilepsy |

**Question 4:**

What physical examinations would you perform on Mrs X?

**Q4 responses (select many):**

| Body height |
| --- |
| Body weight |
| Blood pressure |
| Temperature |
| Respiratory rate |
| Palpate abdomen (Leopold manoeuvres) |
| Listen to fetal heartbeat |
| Pelvic examination to check for oedema/swelling |
| Measure size of womb |
| HEENT |
| Examine lower extremities |

**Question 5:**

What investigations would you perform on Mrs X?

**Q5 responses (select many):**

| Pregnancy test |
| --- |
| Haemoglobin test |
| Urine test for diabetes |
| Malaria test |
| Urine protein |
| Ultrasound |
| Blood platelets count |
| Liver enzymes |
| Serum urea and creatine |
| HIV test |
| STI test |
| Rubella antibodies |
| Blood group and RH and cross matching |
| Hepatitis B or C antigen |
| VDRL or RPR |

**Question 6:**

What drugs or supplies would you provide/ prescribe to Mrs X?

**Q6 responses (select many):**

| Insecticide treated mosquito net |
| --- |
| Iron/folic acid supplements |
| Intermittent preventive treatment for malaria |
| Tetanus vaccination |
| Deworming |

# Supplementary file 3: Coding tree for qualitative analyses

**Factor 1: Helping others and reaching personal goals**

- Altruism as source of wellbeing/pride
- Job as mechanism of community service
- Target setting and support to reach them

**Factor 2: Pride and self-efficacy in job**

- Pride in abilities to give good care
- Barriers to provision of good care
- Absence of materials to do role
- Burnout and impact on work

**Factor 3: External recognition and support (financial and managerial)**

- Salary and financial incentives (within job and in comparable roles)
- Comparability of salary and perceived value
- Community satisfaction and approval
- Fault finding and poor supervision
- Monitoring

**Impact of QI**

- Impact on confidence and self-perception
- Impact on self-efficacy and ability to give good care
- Impact on communication and relationships with peers/managers
- Workload and ability to contribute

# Supplementary table S1: Item response theory (IRT) analyses of health worker knowledge

|  | **(1)**  **History: Previous pregnancies** |  | **(2)**  **History: Current pregnancy** |  | **(3)**  **History: Medical history** |  | **(4)**  **Examinations** |  | **(5)**  **Investigations** |  | **(6)**  **Drugs/ supplies** |  | **(7)**  **Sum of correct knowledge items across all domains** | |
| --- | --- | --- | --- | --- | --- | --- | --- | --- | --- | --- | --- | --- | --- | --- |
| *Panel A: All patient facing staff (HEWs and care providers)* | | | | | | | | | | | | |  |  |
| QI*Endline (*Β*_3_) | 0.25*** | (0.092) | -0.42*** | (0.12) | -0.095 | (0.10) | 0.22* | (0.13) | -0.085 | (0.12) | 0.25*** | (0.075) | 0.31** | (0.129) |
| QI woreda | -0.089 | (0.061) | 0.31*** | (0.080) | -0.011 | (0.061) | -0.059 | (0.081) | 0.061 | (0.097) | -0.11*** | (0.034) | -0.14** | (0.083) |
| Endline | 0.10* | (0.058) | 0.20** | (0.083) | -0.29*** | (0.077) | -0.00065 | (0.084) | -0.093 | (0.091) | 0.28*** | (0.067) | 0.13 | (0.089) |
| Constant | -0.068 | (0.047) | -0.16*** | (0.045) | 0.18*** | (0.047) | -0.023 | (0.063) | 0.037 | (0.071) | -0.14*** | (0.032) | -0.07 | (0.058) |
|  |  |  |  |  |  |  |  |  |  |  |  |  |  |  |
| Observations | 799 |  | 799 |  | 799 |  | 799 |  | 799 |  | 799 |  | 799 |  |
| R-squared | 0.044 |  | 0.022 |  | 0.065 |  | 0.010 |  | 0.009 |  | 0.240 |  | 0.034 |  |
| *Panel B: Midlevel providers* | | | | | | | | | | | | |  |  |
| QI*Endline (*Β*_3_) | 0.38** | (0.18) | -0.36* | (0.20) | -0.094 | (0.17) | 0.37 | (0.23) | -0.069 | (0.14) | 0.15 | (0.13) | 0.31 | (0.200) |
| QI woreda | -0.22* | (0.11) | 0.41*** | (0.11) | 0.079 | (0.093) | -0.23* | (0.13) | 0.060 | (0.10) | -0.12** | (0.057) | -0.26** | (0.101) |
| Endline | 0.087 | (0.13) | 0.11 | (0.14) | -0.43*** | (0.13) | -0.063 | (0.15) | -0.098 | (0.095) | 0.39*** | (0.11) | 0.23 | (0.140) |
| Constant | 0.16* | (0.086) | -0.41*** | (0.063) | -0.039 | (0.074) | 0.38*** | (0.100) | -0.52*** | (0.067) | -0.16*** | (0.055) | 0.46*** | (0.063) |
|  |  |  |  |  |  |  |  |  |  |  |  |  |  |  |
| Observations | 314 |  | 314 |  | 314 |  | 314 |  | 314 |  | 314 |  | 314 |  |
| R-squared | 0.061 |  | 0.030 |  | 0.100 |  | 0.017 |  | 0.011 |  | 0.239 |  | 0.068 |  |
| *Panel C: HEWs* | | | | | | | | | | | | |  |  |
| QI*Endline (*Β*_3_) | 0.20* | (0.12) | -0.54*** | (0.15) | -0.11 | (0.11) | 0.17 | (0.15) | -0.11 | (0.15) | 0.34*** | (0.081) | 0.38** | (0.154) |
| QI woreda | 0.0001 | (0.078) | 0.28** | (0.11) | -0.095 | (0.071) | 0.039 | (0.11) | 0.063 | (0.095) | -0.099** | (0.039) | -0.06 | (0.101) |
| Endline | 0.10 | (0.065) | 0.36*** | (0.093) | -0.21*** | (0.072) | -0.0065 | (0.10) | -0.040 | (0.099) | 0.24*** | (0.071) | -0.01 | (0.086) |
| Constant | -0.25*** | (0.061) | -0.016 | (0.063) | 0.38*** | (0.052) | -0.32*** | (0.086) | 0.44*** | (0.075) | -0.17*** | (0.037) | -0.47*** | (0.071) |
|  |  |  |  |  |  |  |  |  |  |  |  |  |  |  |
| Observations | 411 |  | 411 |  | 411 |  | 411 |  | 411 |  | 411 |  | 411 |  |
| R-squared | 0.043 |  | 0.035 |  | 0.066 |  | 0.014 |  | 0.008 |  | 0.296 |  | 0.037 |  |

# Supplementary table S2: Difference-in-difference estimates of motivation changes by cadre

|  | **Difference-in-difference coefficient (QI*Endline)** | **SE** | **p-value** | **Observations** | **R-squared** |
| --- | --- | --- | --- | --- | --- |
| ***Panel A: Highly skilled care providers*** |  |  |  |  |  |
| Overall motivation | 0.23 | 0.35 | 0.72 | 314 | 0.288 |
| Factor 1 – Helping others and reaching personal goals | -0.2 | 0.13 | 0.89 | 313 | 0.379 |
| Factor 2 – Pride and self-efficacy in job | -0.18 | -0.17 | 0.31 | 310 | 0.427 |
| Factor 3 – External recognition and support (financial and managerial) | -0.07 | 0.24 | 0.68 | 313 | 0.420 |
| ***Panel B: HEWs*** |  |  |  |  |  |
| Overall motivation | -0.07 | 038 | 0.85 | 411 | 0.607 |
| Factor 1 – Helping others and reaching personal goals | -0.10 | 0.22 | 0.63 | 409 | 0.549 |
| Factor 2 – Pride and self-efficacy in job | -0.32 | 0.27 | 0.24 | 407 | 0.613 |
| Factor 3 – External recognition and support (financial and managerial) | -0.15 | 0.2 | 0.46 | 409 | 0.624 |
| ***Panel C: Non-patient-facing staff*** |  |  |  |  |  |
| Overall motivation | -0.01 | 1.18 | 1 | 76 | 0.733 |
| Factor 1 – Helping others and reaching personal goals | -0.1 | 1.43 | 0.95 | 75 | 0.769 |
| Factor 2 – Pride and self-efficacy in job | 0.63 | 1.49 | 0.68 | 76 | 0.763 |
| Factor 3 – External recognition and support (financial and managerial) | -0.2 | 1.01 | 0.84 | 75 | 0.824 |

*Table 5 notes: Each row shows the results from a different regression where each of “overall motivation” and factors 1, 2, and 3 were set as the dependent variable. Overall motivation is a five-level categorical variable where 1=excellent, 2=very good, 3=good, 4=air, 5=poor. Factors 1, 2, and 3 are also bounded between 1 and 5, and are the unweighted mean scores of the constituent variables of each factor, where agreement with statements was classified as 1=strongly agree, 2=agree, 3=neutral, 4=disagree, and 5=strongly disagree. Only the difference-in-difference coefficient* $\beta_{3}$ *from equation (1) is shown, representing (QI woreda*endline). Models were estimated with robust standard errors clustered at facility level*. * *denotes p<0.1.*

# Supplementary figure S1: Scree plot of baseline exploratory factor analysis eigenvalues

# Supplementary figure S2: Plots of Likert scale responses to all variables


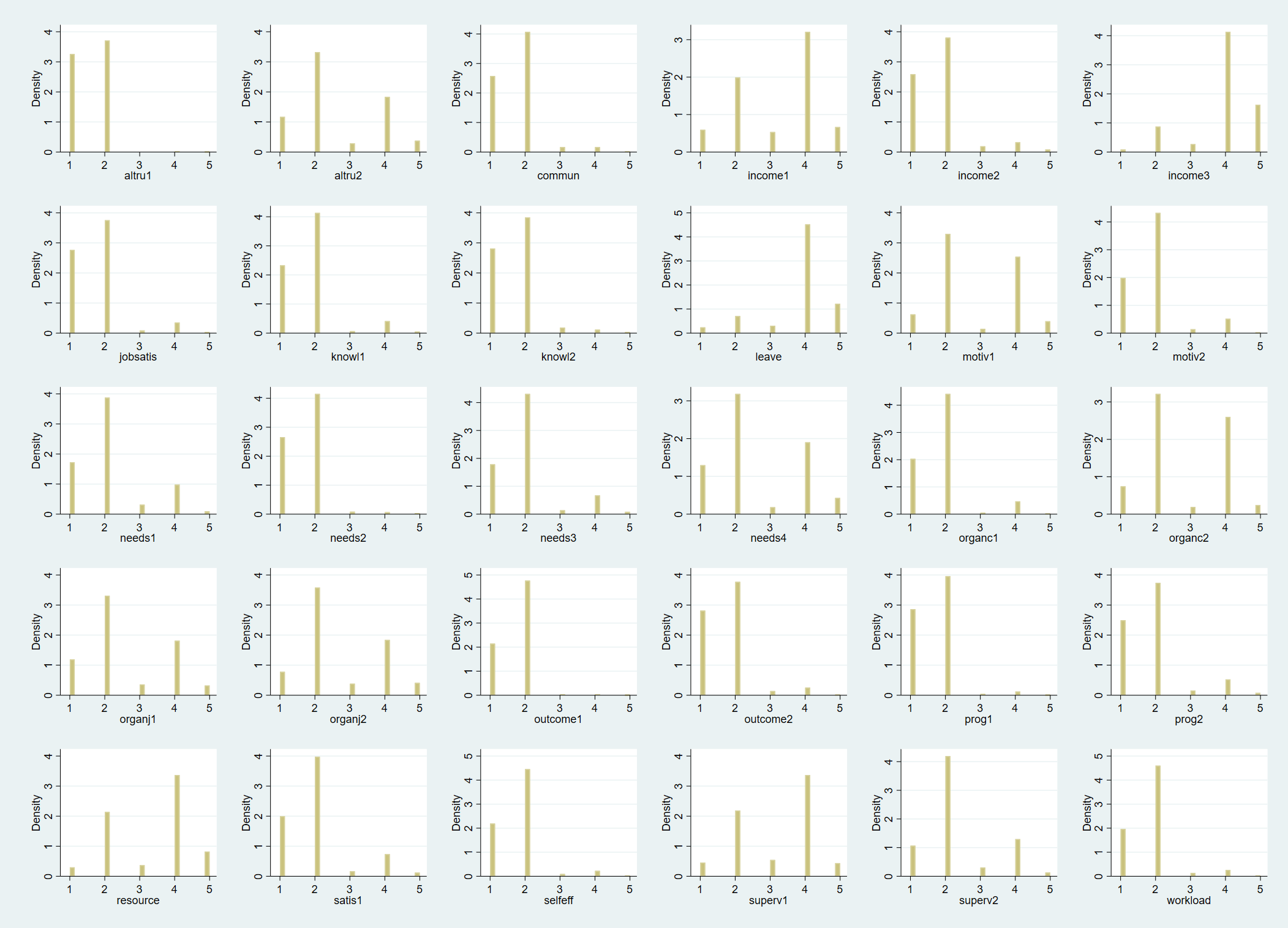


# Supplementary figure S3: Plot of all variables by time point and QI/comparison woreda

# Supplementary figure S4: Change in mean factor scores at baseline and endline, by QI/comparison area


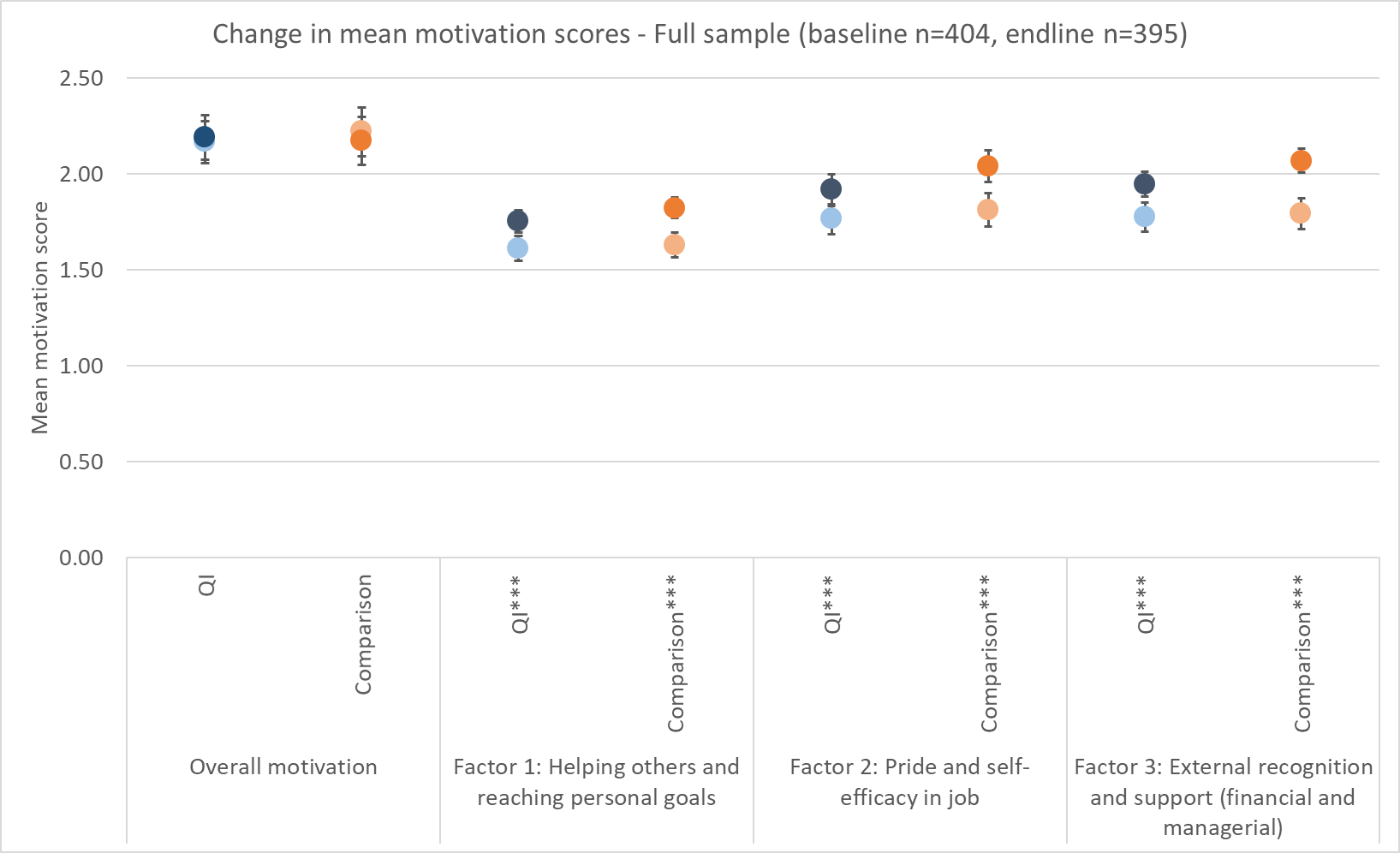


#

Supplementary figure S5: Summary of matched intervention/comparison woreda

*Figure 5 notes: Y axes differ across panels. Figures show average difference across DHS utilisation indicators between all woreda and the intervention woreda, with the closest woreda selected for comparison highlighted in red.* *The Y axis illustrates the average difference in the proportion of DHS respondents reporting utilisation across the three indicators across the three DHS surveys used.*
